# Study on Serological Markers and Brain Structural Changes in Early Clinical Stage of Alzheimer's Disease in Cold Regions

**DOI:** 10.62641/aep.v53i4.1936

**Published:** 2025-08-05

**Authors:** Changhao Yin, Lingyu Chen, Jianhang Wang, Weina Zhao

**Affiliations:** ^1^Department of Neurology, Hongqi Hospital Affiliated to Mudanjiang Medical College, 157000 Mudanjiang, Heilongjiang, China; ^2^Center for Mudanjiang North Medicine Resource Development and Application Collaborative Innovation, 157000 Mudanjiang, Heilongjiang, China

**Keywords:** Alzheimer's disease, cold climate, magnetic resonance imaging, amyloid β protein, microRNA, apolipoproteins E

## Abstract

**Background:**

Subjective cognitive decline (SCD) and mild cognitive impairment (MCI) represent early clinical manifestations of Alzheimer's disease (AD). Recent research has highlighted serum markers and changes in brain structure as promising tools for diagnosing cerebral disorders. This study investigated serum biomarkers and brain structural changes in the early clinical stage of AD affected individuals residing in a cold region.

**Methods:**

Clinical data from patients with SCD or MCI and from normal controls, who were tested at Hongqi Hospital Affiliated to Mudanjiang Medical College from January 2018 to December 2023, were retrospectively analysed. According to clinical classification, the patients were categorised into SCD (*n *= 60), MCI (*n *= 60) and normal control groups (*n *= 70). The magnetic resonance imaging data, serum levels of amyloid β 1-40/42, exosomal *miRNA* (*34a*/*34c*/*135a*) and *apolipoprotein E* (*ApoE*) genotype were collected and analysed.

**Results:**

The mean diffusivity values in the bilateral parahippocampal gyrus, inferior longitudinal bundle, right inferior fronto-occipital tract and posterior cingulate gyrus in the SCD group decreased relative to those of the MCI group (all *p* < 0.05). Conversely, the fractional anisotropy values in the bilateral parahippocampal gyrus, inferior fronto-occipital tract, inferior longitudinal tract and posterior cingulate gyrus in the SCD group increased (all *p* < 0.05). Compared with the normal control group, the MCI and SCD groups showed elevated levels of serum Aβ1-40 and Aβ1-42 and exosomal *miRNA-34a* and *miRNA-34c* (all *p* < 0.05) and decreased exosomal *miRNA-135a* expression (*p* < 0.05). The serum levels of Aβ1-40, Aβ1-42 and exosomal *miRNA-34a* and *miRNA-34c* in the SCD group were lower than those in the MCI group (all *p* < 0.05), whereas *miRNA-135a* level was higher (*p* < 0.05). The proportions of *ApoE* ε*3/3* in the normal control group was the highest (62.86%), and the proportions of *ApoE ε2/4*, *ε3/4* and *ε4/4* in the MCI group were the highest (38.33%, 26.67% and 10.00%, respectively).

**Conclusion:**

Changes in brain structure and serum biomarkers (*miRNAs* and Aβ) are evident in the early stages of AD, and the proportion of *ApoE* alleles vary in early AD. These findings may contribute to the development of an early recognition model for AD.

## Introduction

Alzheimer’s disease (AD) is a degenerative disease of the central nervous system, characterised by progressive cognitive dysfunction and behavioural impairment. It predominantly affects older adults, and its incidence is increasing annually [1]. Epidemiological data indicate that by the end of 2021, the total number of patients with AD and related dementias in China had reached 56.85 million and the prevalence is projected to increase by 60% by 2040 [2]. The long and insidious onset of AD complicates its effective treatment because affected individuals are diagnosed only after substantial neuronal degeneration has already occurred [3]. Therefore, intervention in the early clinical stage of AD is of positive significance for relieving clinical symptoms and controlling the progression of AD disease. Subjective cognitive decline (SCD) and mild cognitive impairment (MCI), as early clinical symptoms of AD [4], have become key areas of interest in current research. Diagnostic approaches for AD often rely on imaging and cerebrospinal fluid detection [5]. Resting-state functional magnetic resonance imaging (rs-fMRI) detects changes in blood oxygen level–dependent signals in the resting state of patients, facilitating analysis of brain activity and functional connectivity. However, rs-fMRI data analysis requires advanced expertise and is complicated by high data heterogeneity [6]. In addition, cerebrospinal fluid analysis is invasive and thus unsuitable for early AD screening [7]. Therefore, the development of a convenient and non-invasive diagnostic method is of great important for early AD screening.

The combination of blood markers and imaging has demonstrated considerable advantages in the early mass screening of diseases. Blood is easier to access than cerebrospinal fluid. Amyloid β (Aβ) is a widely used biomarker in clinical evaluation of AD. It gradually aggregates into soluble oligomers and eventually forms insoluble amyloid plaques. The deposition of these plaques in the cerebral cortex and hippocampus is one of the pathological characteristics of AD [8]. Exosomal *microRNA* (*miRNA*) not only facilitates short-range intercellular communication but also can transmit signals to the brain through the cerebrospinal fluid. Exosomal *miRNAs* are closely related to the pathological process of AD, including participation in the production and clearance of Aβ, neuroinflammatory response and tau protein phosphorylation [9]. The *apolipoprotein E* (*ApoE*) genotype is closely related to the pathological mechanism of AD, influencing Aβ plaque aggregation and clearance, synaptic plasticity and neuroinflammation [10]. Notably, individuals carrying the *ApoE ε4* allele exhibit poorer cognitive function than those with the *ApoE ε2* allele [10]. Despite these findings, rigorous clinical studies on the expression significance of Aβ, exosomal *miRNA* of peripheral blood and *ApoE* in the early stage of AD are still lacking.

The integrity of white matter fibres is impaired in patients with AD because of axonal degeneration and demyelination. These changes are strongly associated with cognitive decline, especially impairments in memory and executive function [11]. Diffusion tensor imaging (DTI) provides a microstructural characterisation of the white matter fibers by detecting the diffusion of water molecules in tissues, especially along white matter fibers [12, 13]. The main indexes of DTI include fractional anisotropy (FA) and mean diffusivity (MD). FA reflects the structural integrity of the fibre, and MD correlates with cell density and structure [12]. Previous study found that FA and MD values in AD patients displayed significant changes in multiple brain regions [13].

In recent years, the impacts of temperature and climate on the AD have attracted considerable attention. Exposure to extreme heat has been associated with increased hospitalization rate in patients with AD in the USA [14]. A cross-sectional survey reported that exposure to extreme cold for over 2 days is related to cognitive decline in individuals residing mediterranean or oceanic climates, and cognitive performance in older people was found to fluctuate with seasonal changes [15]. In China, low temperatures have been associated with increased mortality rate in patients with AD [16]. Despite these findings, no study has explored changes in brain structure and serum metabolites of in patients in early clinical stages of AD in cold areas. Therefore, this study aimed to explore the relationship between serological markers and changes in brain structure in patients with early-stage AD in cold areas. Retrospective analysis was conducted on the clinical and imaging data of patients with AD at various stages in Heilongjiang Province. The finding may provide important insights into the role of serological markers in the pathogenesis of AD in cold regions.

## Patients and Methods

### Research Object

This study retrospectively analysed the clinical data of patients with early-stage AD and healthy people treated in Hongqi Hospital Affiliated to Mudanjiang Medical College from January 2018 to December 2023. The subjects were divided into SCD (*n* = 60), MCI (*n* = 60) and normal control groups (*n* = 70) according to clinical classification. The study adhered to the principles of the Declaration of Helsinki and has received approval from the ethics committee Hongqi Hospital Affiliated to Mudanjiang Medical College (Approval No. 202311). Informed consent was obtained from patients and their legal guardians.

### Inclusion and Exclusion Criteria

All subjects were of Han nationality and right-handed. Each subject underwent a series of neuropsychological assessments, including the Mini-mental State Examination (MMSE) [17], the Auditory Verbal Learning Test (AVLT) [18], the Montreal Cognitive Assessment Form (MoCA) [19] and the Clinical Dementia Rating Scale (CDR) [20].

#### Inclusion Criteria for the Normal Control Group

The inclusion criteria for the normal control group were as follows:

(1) age ≥60 years old;

(2) no complaint of cognitive decline;

(3) cognitive function tests within the normal range;

(4) matched with the SCD and MCI groups in terms of age, gender and education level;

(5) no positive signs and symptoms in neurological examination and medical examinations.

#### Inclusion Criteria for Patients With SCD

According to the 2014 SCD diagnostic framework criteria [21]:

(1) age ≥60 years;

(2) onset time within 5 years;

(3) complaints of memory loss, supported by corroborative evidence from relatives;

(4) CDR score of 0;

(5) normal performance in general relevant cognitive tests.

#### Inclusion Criteria for Patients With MCI

According to the 2014 Sachdev PS diagnostic framework criteria [22]:

(1) age ≥60 years;

(2) complaints of memory loss, confirmed by relatives;

(3) cognitive decline in one or more domains but normal or slightly impaired abilities for daily living;

(4) memory decline indicated by AVLT tests;

(5) did not meet the diagnostic criteria for AD;

(6) CDR score of 0.5;

(7) cognitive decline due to other diseases.

#### Exclusion Criteria

Participants were excluded if they met any of the following criteria:

(1) diagnosis of neurological disorders known to impair cognitive function, such as Parkinson’s disease, vasogenic dementia and progressive supranuclear palsy;

(2) history of neurological damage related to multiple sclerosis, brain trauma and other diseases;

(3) brain structural abnormalities;

(4) magnetic resonance imaging (MRI) showing infarction, infection, focal injury and multiple lacunar infarction;

(5) contraindications to MRI, such as metallic foreign bodies, including pacemakers, aneurysm clips, artificial heart valves and metal fragments;

(6) schizophrenia, bipolar disorder and other mental diseases;

(7) history of alcohol or drug abuse or addiction within 2 years;

(8) incomplete clinical data.

#### Neuropsychological Scales

The CDR is a semi-structured interview tool wildly used to assess the severity of dementia. It generates a comprehensive dementia score by evaluating performance across multiple cognitive and functional domains: memory, orientation, judgment and problem solving, community affairs, home and hobbies and personal care. The score for each field ranges from 0 to 3: 0 (normal), 0.5 (questionably or minimally impaired), 1 (mildly impaired), 2 (moderately impaired) and 3 (severely impaired). The total score is 0–18 points. The sensitivity and specificity of the CDR for MCI were 93% and 97%, respectively [20].

The MMSE is commonly used to assess cognitive function. It contains 11 items that cover various areas of cognitive domains: orientation, memory, attention and calculation, language and visual construction. It has a total score of 30 points. The education-specific cutoff points of cognitive impairment are as follows: ≤19, illiterate; ≤22, with elementary school education; and ≤26, with middle school education and above [17]. The sensitivity of MMSE can reach 97.1% for dementia [17].

The AVLT can evaluate learning and memory abilities. It consists of four stages: (1) Immediate recall (0–36 points): after reading 12 words, participants are asked to immediately recall as many words as possible. The test is repeated three times, and the reading order of the words does not change on each test. The score of participants is the total number of words recalled correctly on three tests. (2) Short-term delayed recall (0–12 points): after 5 min, participants are asked to recall the 12 words freely. (3) Long-term delayed recall (0–12 points): after 20 min, participants are asked to recall the words. (4) Recognition (0–24 points): after long-term delay recall, a recognition test is performed. The test includes 12 target and 12 distractor words, and participants are asked to distinguish between target and distractor words. High scores represent enhanced memory and learning abilities [18].

The MoCA is used to assess cognitive impairment and has a total score of 30. The scale contains 11 items: address orientation, drawing figures, naming objects, processing speed, memory, attention, recall, vigilance, repetition, verbal fluency and abstraction. If years of education is ≤12 years, the MoCA adds one point, but the total score cannot exceed 30 points. The cutoff points of cognitive impairment according to education are as follows: ≤19, 6 years of education of less; ≤22, 7–12 years of education; and ≤24, more than 12 years of education. The Cronbach’s α of the MoCA scale is 0.807 [19].

### Magnetic Resonance Data Acquisition and Processing

MRI was conducted for all subjects in Hongqi Hospital Affiliated to Mudanjiang Medical College with a Philips Achieva 3.0T MRI scanner (Philips Medical Systems, Best, The Netherlands) equipped with a 20-channel head coil. The subjects were instructed to lie flat and remain relaxed during the procedure. T1-weighted 3D magnetization–prepared rapid acquisition gradient-echo (MP-RAGE) with the following parameters: repetition time (TR), 8.2 ms; echo time (TE), 3.7 ms; field of view (FOV), 256 mm × 256 mm; turning angle, 7°; number of layers, 188; layer thickness, 1 mm; layer spacing, 0 mm; matrix size, 128 mm × 128 mm; and voxel, 1 mm × 1 mm × 1 mm. Subsequently, DTI was performed using a single shot echo planar imaging (EPI) sequence with the following parameters: TR, 8000 ms; TE, 98 ms, b value of 0 and 1000 s/mm^2^; 30 non-collinear directions; FOV, 256 mm × 256 mm; matrix size, 128 mm × 128 mm; layer thickness, 3 mm; and layer spacing, 0 mm.

MRI data from T1-weighted 3D MP-RAGE was pre-processed using Statistical Parametric Mapping 8 (SPM8) (http://www.fil.ion.ucl.ac.uk/spm, Wellcome Trust Centre for Neuroimaging, London, UK) and Data Processing Assistant for Resting-State fMRI (DPARSF) version 4.0 (http://rfmri.org/DPARSF). Images were segmented, aligned, spatially normalised and smoothed. Images with translation of >2 mm and rotation of >2 in any direction were excluded. The images were smoothed with a full width at half maximum of 8 mm. The corrected images were spatially standardised using the standard EPI template of SPM and then resampled, and signals within the frequency range of 0.01–0.08 Hz was extracted using a band-pass filter. Regions of interest (ROIs) were drawn in the brain regions on the T1-weighted images and then registered onto DTI for the analysis of MD and FA.

### Determination of Blood-Related Components

Aβ: Fasting venous blood (5 mL) was collected and centrifuged (3000 r/min, 4 °C, 10 min). The serum was separated, collected and stored at –80 °C. The serum levels of Aβ1-40 and Aβ1-42 were measured according to the operating instructions of the kits (Aβ1-42: SP11457; Aβ1-40: SP11731; Wuhan Saipei Biotechnology Co., Ltd., Wuhan, China).

Exosome *miRNA*: Fasting venous blood (5 mL) was collected from the elbow. Exosomes were extracted from blood samples with an exosome extraction and purification kit (UR52151, Umibo Biotechnology, Shanghai, China) according to the operating instructions. Trizol reagent (Solarbio, Beijing, China) was used to extract total RNA from exosomes, and Nanodrop was used to detect the purity and concentration of total RNA. Total RNA was reverse transcribed into cDNA with a reverse transcription kit (TaKaRa, Dalian, China), and the relative expression levels of exosomal *miRNA-34a*, *miRNA-34c* and *miRNA-135a* were detected through quantitative reverse transcriptase polymerase chain reaction (qRT-PCR). The standard three-step method was used for qRT-PCR detection. First, the pre-denaturation reaction was performed at 95 °C for 5 min, followed by denaturation at 95 °C for 10 s, annealing at 60 °C for 20 s and extension at 72 °C for 15 s. A total of 40 cycles of detection were required. The *U6* was used as an internal inference, and the 2^–Δ⁢Δ⁢Ct^ method was used for analysis. The primers were provided by Ribobio (Guangzhou, China), and the sequences are presented in Table 1. The downstream primers of *miRNA* were universal primers and were designed by the stem-ring method. Given that *miRNA* sequences were short, the length of each *miRNA* reverse transcript was first extended with a stem-ring sequence during reverse transcription for subsequent PCR procedures. Downstream primers were intercepted from the stem-ring sequence and did not cover the *miRNA* sequences.

**Table 1.** **Primer sequences for quantitative reverse transcriptase real-time polymerase chain reaction (qRT-PCR)**.

Primers	Sequence (5′-3′)
*miRNA-34a*	Forward: ACACTCCAGCTGGGTGGCAGTGTCTTAGC
	Reverse: CTCAACTGGTGTCGTGGAGTC
*miRNA-34c*	Forward: ACACTCCAGCTGGGAATCACTAACCACACG
	Reverse: CTCAACTGGTGTCGTGGAGTC
*miRNA-135a*	Forward: ACACTCCAGCTGGGTATGGCTTTTTATTCC
	Reverse: CTCAACTGGTGTCGTGGA
*U6*	Forward: GCTTCGGCAGCACATATACTAAAAT
	Reverse: CGCTTCACGAATTTGCGTGCAT

### Identification of ApoE Genotype

Fasting venous blood (5 mL) was collected, and genomic DNA was extracted using a genomic DNA extraction kit (QIAGEN, Hilden, NRW, Germany) for gene amplification. The amplified products were digested with Hhal enzyme (GCGC) and then subjected to agarose gel electrophoresis. *ApoE* alleles were determined by imprinting hybridisation.

### Statistical Analysis

Statistical processing and analysis were performed using SPSS 26 software (IBM, Armonk, NY, USA). The Shapiro–Wilk test was applied for the detection of normal distribution. The normally distributed quantitative data were presented as mean ± standard deviation. Differences among the three groups were assessed using one-way analysis of variance with Tukey’s post hoc test. The non-normally distributed quantitative data were expressed as median (quartiles) and compared using the Kruskal–Wallis test with Dunn’s post hoc analysis. Categorical data were analysed using the chi-square test (χ^2^ test). All statistical tests were two sided, and a *p* value of less than 0.05 was considered statistically significant.

## Results

### General Information

A total of 190 subjects were included in the study and divided into SCD (60 cases), MCI (60 cases) and normal control groups (70 cases) according to their disease types. The general information of all subjects is shown in Table 2. No significant differences in gender, age, body mass index, years of education, education level and comorbid diseases were observed among three groups (all *p* > 0.05). The MMSE, MoCA and AVLT scores decreased (all *p* < 0.05) relative to those in the normal control and SCD groups. This result indicated a decline in cognitive function and memory performance in the MCI group.

**Table 2.** **General information**.

Items		Normal control group (*n* = 70)	MCI group (*n* = 60)	SCD group (*n* = 60)	*F/H/χ^2^*	*p*
Gender (male/female)		41/29	35/25	34/26	0.055	0.973
Age (years)		70.01 ± 3.47	68.78 ± 5.28	69.18 ± 4.71	1.277	0.281
BMI (kg/m^2^)		24.72 ± 3.32	24.82 ± 3.09	25.18 ± 2.90	0.378	0.686
Years of Education (years)		7 (5, 13)	11 (5, 13)	8 (5, 14)	0.084	0.959
Education level (n, %)					1.650	0.949
	Illiterate	7 (10.0%)	5 (8.3%)	8 (13.3%)		
	Primary school	29 (41.4%)	21 (35.0%)	22 (36.7%)		
	Middle school	22 (31.4%)	23 (38.3%)	20 (33.3%)		
	University or above	12 (17.1%)	11 (18.3%)	10 (16.7%)		
Hypertension (n, %)		25 (35.7%)	17 (28.3%)	15 (25.0%)	1.882	0.390
Hyperlipidaemia (n, %)		15 (21.4%)	11 (18.3)	9 (15.0%)	0.889	0.641
Coronary heart disease (n, %)		12 (17.1%)	8 (13.3%)	14 (23.3%)	2.085	0.353
Diabetes (n, %)		22 (31.4%)	19 (31.7%)	18 (30.0%)	0.046	0.977
MMSE score		28 (27, 29)	23 (20, 24)*	27 (26, 28)^&^	100.359	<0.001
MoCA score		27 (27, 28)	20 (18, 22)*	26 (23, 27)*^&^	131.563	<0.001
AVLT score						
	Immediate Recall	22.61 ± 3.47	16.62 ± 2.78*	21.50 ± 3.56^&^	58.438	<0.001
	Short-term Delayed recall	6.59 ± 1.11	4.95 ± 1.27*	6.02 ± 1.08*^&^	33.169	<0.001
	Long-term Delayed recall	6.08 ± 1.10	4.03 ± 1.35*	6.00 ± 1.16^&^	57.596	<0.001
	Recognition	21.23 ± 2.02	17.33 ± 1.43*	20.02 ± 1.17*^&^	97.900	<0.001

Note: MCI, mild cognitive decline; SCD, subjective cognitive decline; MMSE, mini-mental state examination; BMI, body mass index; CDR, Clinical Dementia Rating Scale; MoCA, Montreal Cognitive Assessment Form; AVLT, Auditory Verbal Learning Test. *p < 0.05 normal control group versus MCI or SCD group; ^&^*p* < 0.05 MCI group versus SCD group.

### Comparison of MRI Examination Results

**Fig. 1.**
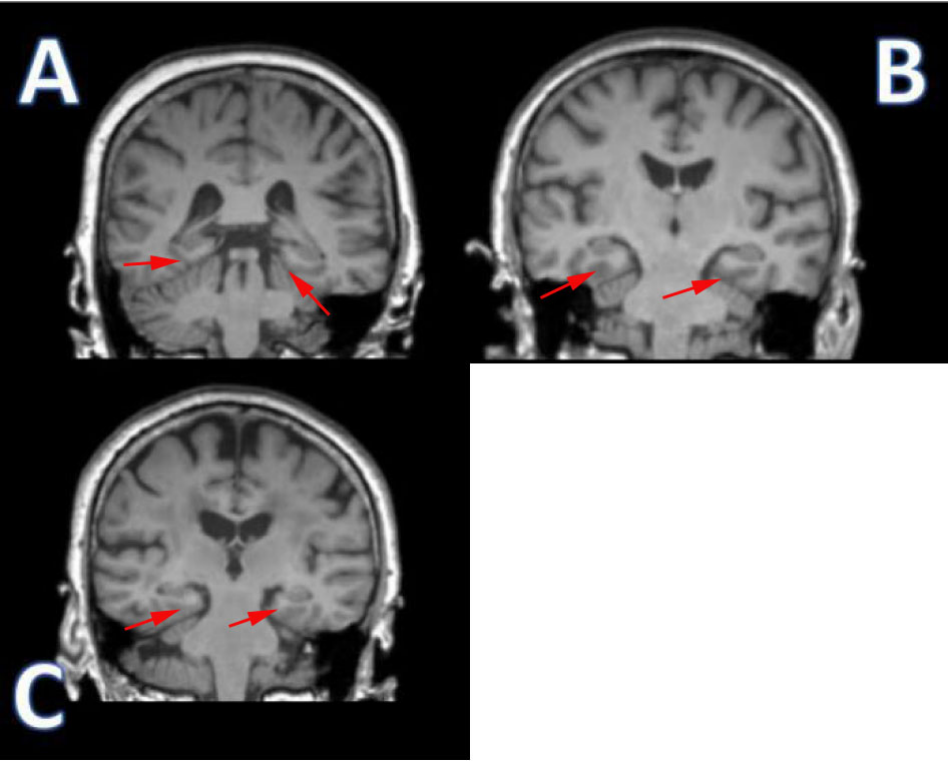
**Different changes in coronal magnetic resonance imaging of the parahippocampal gyrus in the three groups**. (A) Normal control: the area indicated by the red arrows show the normal structure of parahippocampal gyrus, without significant atrophy or abnormal signal. (B) Subjective cognitive decline (SCD): the area indicated by the red arrows show a slight decrease in gray matter volume, and a slight blurring of the outline of the parahippocampal gyrus. (C) Mild cognitive impairment (MCI): the area indicated by the red arrows show a marked reduction in gray matter volume. The parahippocampal gyrus is markedly atrophied, with blurred borders and morphological alterations.

**Fig. 2.**
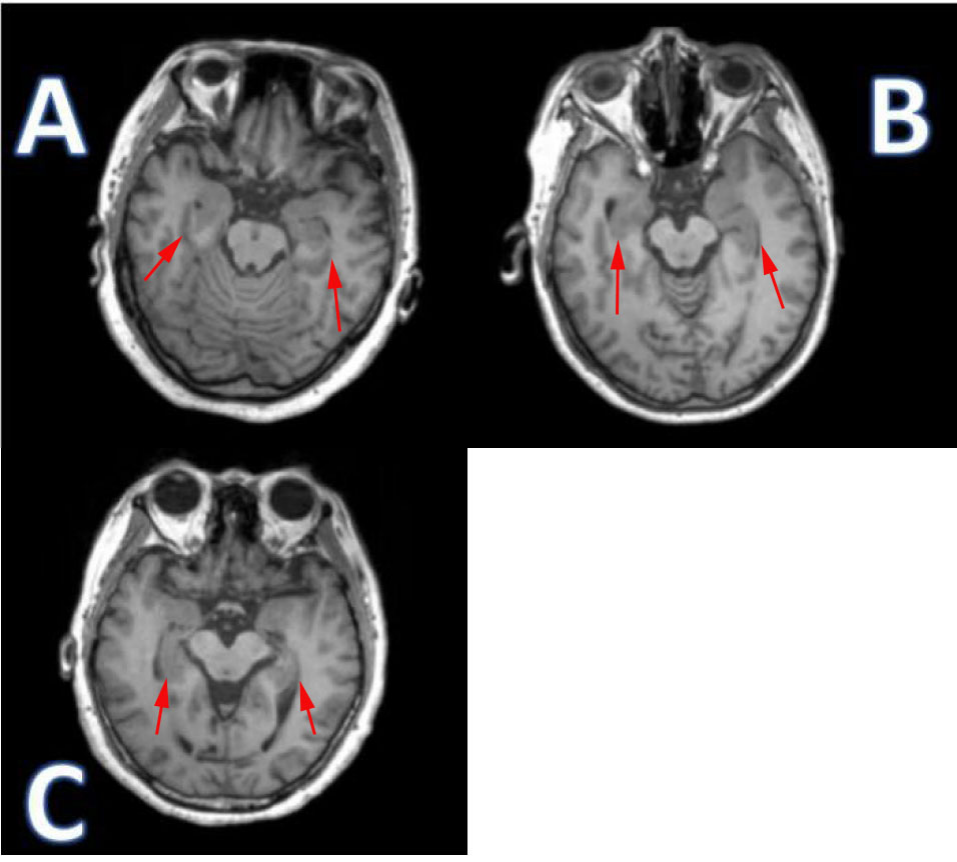
**Different axial changes in magnetic resonance imaging of the parahippocampal gyrus in the three groups**. (A) Normal control: the area indicated by the red arrows show the normal parahippocampal gyrus structure. (B) Subjective cognitive decline (SCD): the area indicated by the red arrows show that the contour of the hippocampus is slightly blurred. (C) Mild cognitive impairment (MCI): the area indicated by the red arrows show the significant atrophy, blurred boundaries and reduced gray matter volume in the parahippocampal gyrus.

**Fig. 3.**
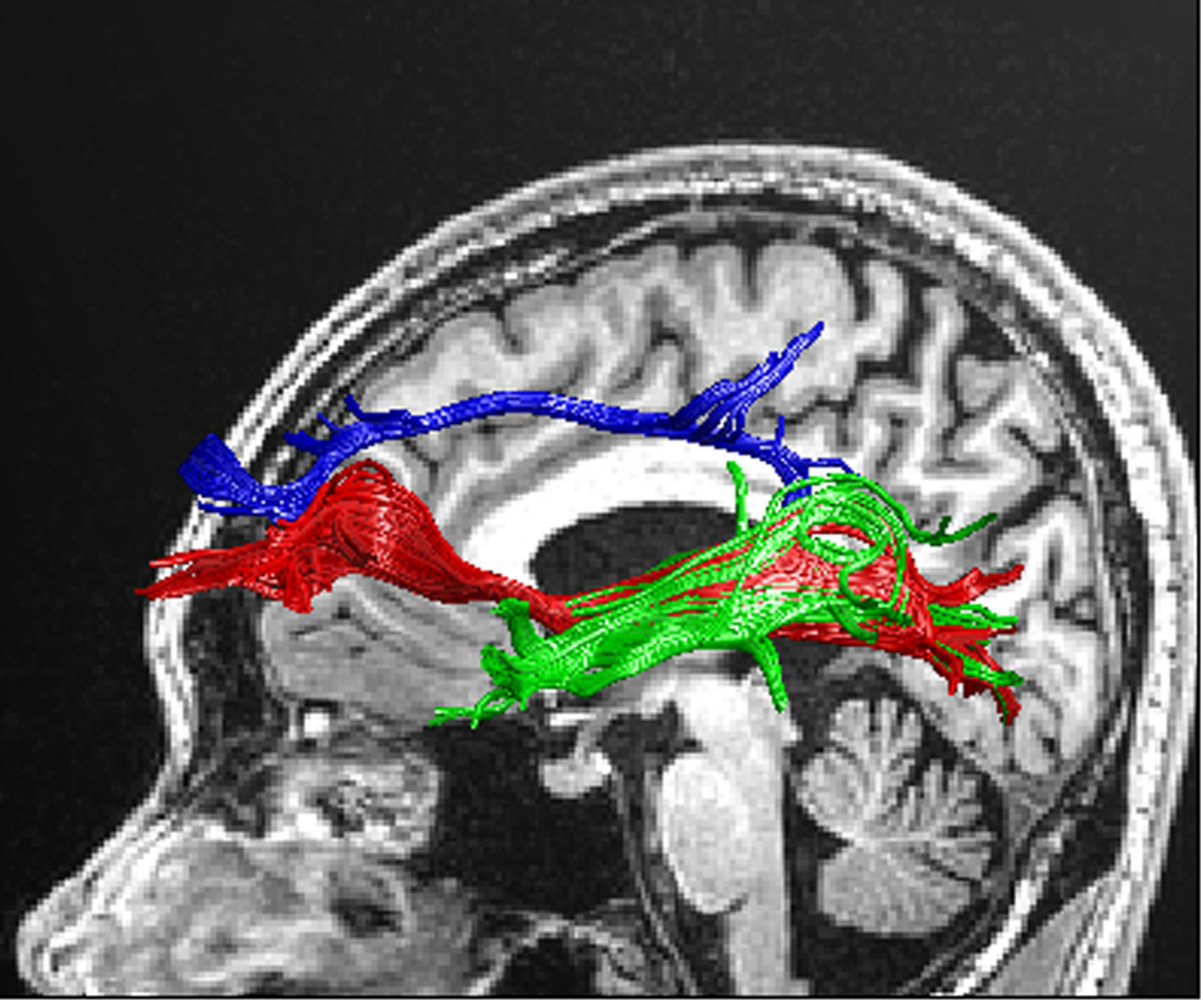
**Schematic of magnetic resonance imaging of the fronto-occipital tract, inferior longitudinal bundle and posterior cingulate gyrus**. Green: fronto-occipital tract; blue: inferior longitudinal bundle; red: posterior cingulate gyrus.

Compared with normal control group, the MCI and SCD groups showed significant increases in the MD values of bilateral parahippocampal gyrus, inferior fronto-occipital tract and left posterior cingulate gyrus (all *p* <0.05) and significant decreases in the FA values of bilateral parahippocampal gyrus, left inferior fronto-occipital tract, bilateral inferior longitudinal bundle and bilateral posterior cingulate gyrus (all *p* < 0.05). The MD values of the bilateral inferior longitudinal bundle and right posterior cingulate gyrus in the MCI group were higher than those in the normal control group (all *p* < 0.05), whereas the FA value of right inferior fronto-occipital tract in the MCI group was lower than that in the normal control group (*p* < 0.05). Compared with the MCI group, the SCD groups showed significant decreases in the MD values of bilateral parahippocampal gyrus and inferior longitudinal bundle and right inferior fronto-occipital tract and posterior cingulate gyrus (all *p* < 0.05) and significant increases in the FA values of bilateral parahippocampal gyrus, inferior fronto-occipital tract, inferior longitudinal tract and posterior cingulate gyrus (all *p* < 0.05; Table 3; Figs. 1,2,3).

**Table 3.** **Comparison of MRI results**.

Items		Normal control group (*n* = 70)	MCI group (*n* = 60)	SCD group (*n* = 60)	*F*	*p*
MD value	Parahippocampal gyrus (right)	7.39 ± 0.75	8.12 ± 0.73*	7.73 ± 0.79*^&^	15.034	<0.001
	Parahippocampal gyrus (left)	7.08 ± 0.69	8.61 ± 0.71*	7.66 ± 0.79*^&^	71.744	<0.001
	Inferior fronto-occipital tract (right)	7.30 ± 0.81	8.59 ± 0.75*	8.09 ± 0.64*^&^	50.400	<0.001
	Inferior fronto-occipital tract (left)	7.41 ± 0.66	7.91 ± 0.60*	7.81 ± 0.71*	10.675	<0.001
	Inferior longitudinal bundle (right)	7.25 ± 0.81	8.34 ± 0.79*	7.43 ± 0.77^&^	34.078	<0.001
	Inferior longitudinal bundle (left)	7.31 ± 0.69	8.27 ± 0.71*	7.61 ± 0.78^&^	29.065	<0.001
	Posterior cingulate gyrus (right)	7.05 ± 0.81	7.69 ± 0.77*	7.28 ± 0.79^&^	10.709	<0.001
	Posterior cingulate gyrus (left)	6.58 ± 0.73	7.41 ± 0.81*	7.24 ± 0.68*	23.115	<0.001
FA value	Parahippocampal gyrus (right)	0.59 ± 0.06	0.39 ± 0.03*	0.50 ± 0.03*^&^	340.810	<0.001
	Parahippocampal gyrus (left)	0.55 ± 0.07	0.41 ± 0.05*	0.47 ± 0.06*^&^	86.125	<0.001
	Inferior fronto-occipital tract (right)	0.47 ± 0.03	0.40 ± 0.06*	0.46 ± 0.06^&^	34.323	<0.001
	Inferior fronto-occipital tract (left)	0.53 ± 0.06	0.38 ± 0.05*	0.42 ± 0.05*^&^	136.819	<0.001
	Inferior longitudinal bundle (right)	0.54 ± 0.05	0.42 ± 0.06*	0.49 ± 0.05*^&^	81.913	<0.001
	Inferior longitudinal bundle (left)	0.57 ± 0.06	0.40 ± 0.05*	0.48 ± 0.06*^&^	144.355	<0.001
	Posterior cingulate gyrus (right)	0.56 ± 0.07	0.43 ± 0.06*	0.51 ± 0.05*^&^	73.689	<0.001
	Posterior cingulate gyrus (left)	0.58 ± 0.08	0.41 ± 0.07*	0.50 ± 0.05*^&^	99.418	<0.001

Note: MCI, mild cognitive decline; SCD, subjective cognitive decline; MRI, Magnetic Resonance Imaging; MD, mean diffusivity; FA, fractional anisotropy. *p < 0.05 normal control group versus MCI or SCD group; ^&^*p* < 0.05 MCI group versus SCD group.

### Comparison of Serum Aβ Levels

Significant differences in the serum levels of Aβ1-40, Aβ1-42 and Aβ1-42/Aβ1-40 were observed among the three groups (all *p* < 0.001). Compared with the normal control group, the MCI and SCD groups showed increased serum Aβ1-40 levels (all *p* < 0.05). The Aβ1-42 levels in the MCI group were higher than those in the normal control group (*p* < 0.05). The levels of serum Aβ1-40, Aβ1-42 and Aβ1-42/Aβ1-40 in the SCD group were lower than those in the MCI group (all *p* < 0.05; Table 4).

**Table 4.** **Comparison of serum Aβ related indexes**.

Groups	Aβ1-40 (pg/mL)	Aβ1-42 (pg/mL)	Aβ1-42/Aβ1-40
Normal control group (*n* = 70)	190.35 (177.76, 203.64)	19.43 (17.48, 21.90)	0.10 (0.09, 0.12)
MCI group (*n* = 60)	238.69 (225.00, 253.76)*	28.49 (23.63, 31.94)*	0.12 (0.10, 0.13)
SCD group (*n* = 60)	214.31 (199.35, 229.18)*^&^	19.85 (16.27, 25.11)^&^	0.09 (0.07, 0.12)^&^
*H*	101.724	50.066	15.299
*p*	<0.001	<0.001	<0.001

Note: MCI, mild cognitive decline; SCD, subjective cognitive decline; Aβ, amyloid β. *p < 0.05 normal control group versus MCI or SCD group; ^&^*p* < 0.05 MCI group versus SCD group.

### Comparison of miRNA Expression in Serum Exosomes

The expression levels of serum exosomal *miRNA-34a*, *miRNA-34c* and *miRNA-135a* of the three groups were compared, and the differences were statistically significant (*p* < 0.001, *p* < 0.001, and *p* < 0.001, respectively). Compared with the normal control group, the MCI and SCD groups showed significantly increased *miRNA-34a* and *miRNA-34c* expression levels and decreased *miRNA-135a* expression level (all *p* < 0.05). The expression levels of serum exosomal *miRNA-34a* and *miRNA-34c* in the SCD group were lower, and *miRNA-135a* expression level was higher than that in the MCI group (all *p* < 0.05; Table 5).

**Table 5.** **Expression of exosome *miRNA***.

Groups	Exosome *miRNA*		
	*miRNA-34a*	*miRNA-34c*	*miRNA-135a*
Normal control group (*n* = 70)	1.02 ± 0.10	1.01 ± 0.09	1.00 ± 0.10
MCI group (*n* = 60)	1.96 ± 0.17*	1.84 ± 0.23*	0.64 ± 0.10*
SCD group (*n* = 60)	1.57 ± 0.21*^&^	1.49 ± 0.22*^&^	0.77 ± 0.18*^&^
*F*	544.514	323.928	127.566
*p*	<0.001	<0.001	<0.001

Note: MCI, mild cognitive decline; SCD, subjective cognitive decline; miRNA, microRNA. *p < 0.05 normal control group versus MCI or SCD group; ^&^*p* < 0.05 MCI group versus SCD group.

### Distribution of ApoE Alleles

The results of PCR-multiplex Taqman-MGB probe combination technique showed that six *ApoE* genotypes were detected. Among the three groups, the proportion of *ApoE ε3/3* in the normal control group was the highest (62.86%), and proportions of *ApoE ε2/4*, ε*3/4* and ε*4/4* in the MCI group were the highest (38.33%, 26.67%, and 10.00%, respectively). In the normal control group, *ApoE ε3/3* had the highest proportion (62.86%), followed by *ApoE ε2/3* (22.86%). In the MCI group, the proportion of *ApoE ε2/4* was the highest (38.33%), and *ApoE ε3/4* and ε*3/3* accounted for 26.67% and 13.33%, respectively. For patients with SCD, *ApoE ε3/3* had the highest proportion (31.67%), followed by *ApoE ε2/4* (25.00%) and *ApoE ε3/4* (23.33%; Table 6).

**Table 6.** **Distribution of *ApoE* alleles**.

Groups	*ε* alleles					
	*ε2/2*	*ε2/3*	*ε3/3*	*ε2/4*	*ε3/4*	*ε4/4*
Normal control group (*n* = 70)	3 (4.29%)	16 (22.86%)	44 (62.86%)	4 (5.71%)	3 (4.29%)	0 (0.00)
MCI group (*n* = 60)	0 (0.00)	7 (11.67%)	8 (13.33%)*	23 (38.33%)*	16 (26.67%)*	6 (10.00%)*
SCD group (*n* = 60)	0 (0.00)	11 (18.33%)	19 (31.67%)*^&^	15 (25.00%)*^&^	14 (23.33%)*	1 (1.67%)*
χ ^2^	5.225	2.765	35.074	20.391	13.450	10.111
*p*	0.073	0.251	<0.001	<0.001	0.001	0.006

Note: MCI, mild cognitive decline; SCD, subjective cognitive decline; ApoE, Apolipoprotein E. *p < 0.05 normal control group versus MCI or SCD group; ^&^*p* < 0.05 MCI group versus SCD group.

## Discussion

AD is a progressive and irreversible degenerative disease of the nervous system. The characteristic pathological changes in patients with AD include neurofibrillary tangles formed by hyperphosphorylated tau protein and spots formed by Aβ deposition [23], and changes in pathologic markers and brain structural precede decline in cognitive function in patients with AD [24]. When a clear clinical diagnosis of AD is established, preventive interventions are no longer effective. MCI is a pre-clinical stage of AD and develops into AD in some patients [25]. SCD refers to subjective perceptions of cognitive decline, but objective cognitive test results remain within the normal range. Notably, SCD is considered a pre-clinical manifestation of AD [26]. Therefore, exploring changes in brain structure and the pathological markers of SCD and MCI is essential for the early diagnosis and prevention of AD. This study found that the brain structure of patients with early-stage AD exhibited different MRI characteristics, and the levels of serum Aβ1-40 and Aβ1-42 and exosomal *miRNA-34a* and *miRNA-34c* were higher than those in the normal control group. By contrast, *miRNA-135a* expression levels in these patients were lower than those in the normal control group. Significant differences in the levels of these serum biomarkers were observed between the patients with SCD and MCI. Finally, the distribution of *ApoE*genotypes indicated differences among healthy individuals and patients with SCD and MCI. These results indicated that the patients with SCD and MCI presented with changes in brain structure and serum biomarkers, and the *ApoE* genotype was associated with the progression of early-stage AD. In addition, as all the patients were from a cold region, temperature may be a contributing factor affecting the preclinical progression of AD.

In this study, the MRI indexes of all subjects were analysed. The results showed compared with the normal control group, in MCI and SCD groups showed significantly increased MD values for the bilateral parahippocampal gyrus, inferior fronto-occipital tract and left posterior cingulate gyrus and significantly reduced FA values for the bilateral parahippocampal gyrus, left inferior fronto-occipital tract, bilateral inferior longitudinal bundle and bilateral posterior cingulate gyrus. These findings indicated that the brain structure changed in the pre-clinical stage of AD. Compared with the MCI group, the SCD group showed lower MD values for the bilateral parahippocampal gyrus, inferior fronto-occipital tract, inferior longitudinal tract and posterior cingulate gyrus and higher FA values for the bilateral parahippocampal gyrus, inferior fronto-occipital tract, inferior longitudinal tract and posterior cingulate gyrus. This results multiple brain regions are involved in structural changes associated with the pre-clinical stage of AD.

The FA value can reflect the integrity of white matter fibres, and a high FA value represents nerve conduction capacity. Increased MD values indicate impaired fibre integrity or tissue compactness [12]. Our research found decreased FA values and increased MD values in the bilateral parahippocampal gyrus, inferior fronto-occipital tract, inferior longitudinal bundle and posterior cingulate gyrus in patients with MCI or SCD. This result suggested the degeneration of white matter fibres in these areas and the subsequent reduction in nerve conduction capacity. The white matter connects multiple brain regions and can transmit signals in different brain regions. White matter damage could impede nerve conduction, affecting memory and cognitive functions [27]. Patients with AD present changes in the white matter, including demyelination and function alterations in oligodendrocytes [28]. In addition, damage in white matter microstructure is an early signal of AD [29]. Decreased FA, increased MD and increased radial diffusivity were observed in patients with SCD compared with normal healthy populations [30]. This result is consistent with our fundings. Another study reported the connection between MoCA score and MD value at bilateral inferior cerebellar peduncles and the pyramids segment of right corticospinal tract and between FA value and International Physical Activity Questionnaire-Short Form metabolic equivalent of task (IPAQ-SF MET) in SCD patients [31]. This suggested that FA and MD values may be associated with the pre-clinical progression of AD. In this study, we observed a decrease in FA value and an increase in MD value in patients with MCI compared with patients with SCD. However, we were unable to determine whether FA and MD values can be used in predicting the early progression of AD. This gap needs to be further explored in future studies. In addition, we observed differences in the MD and FA values of multiple brain regions among the normal control and patients with SCD and MCI. Clear thresholds or reference ranges defining the boundaries between normal participants and those with cognitive impairment were not identified. Currently, no clear boundaries or reference values for FA and MD have been established to differentiate normal cognitive function from pathological changes due to a disease. The possible reason is that FA and MD values are not only affected by acquisition procedures and analysis methods but also related to physiological differences between individuals [32]. Such variability complicates the identification of uniform cutoffs points for distinguishing healthy individuals from those with a disease.

Aβ deposition is the main pathological feature of AD and is closely related to the mechanism of neurodegeneration [8]. Aβ1-42 can participate in the pathogenesis of AD by promoting the phosphorylation of tau protein, neuronal death and apoptosis. Meanwhile, Aβ1-40 and Aβ1-42 can aggregate into amyloid fibres [8]. Our study showed that the MCI group had the highest serum Aβ1-40 level, followed by the SCD and normal control groups. The Aβ1-42 levels in the MCI group were higher than those in the normal control group and SCD group. Circulating Aβ proteins enter the brain through the blood–brain barrier and promote the accumulation of amyloid proteins [33]. Meanwhile, peripheral Aβ can activate immune cells and promote the release of cytokines, which enter the brain by crossing the blood–brain barrier and activate microglia and induce neuroinflammation [34]. Increased plasma Aβ1-42 and Aβ1-40 levels had been observed in patients with AD [35], but some studies did not observed differences in plasma Aβ1-42 and Aβ1-40 levels between MCI and normal control groups [36]. This discrepancy may be attributed to difference in detection method used (immunomagnetic reduction). In addition, low Aβ1-42/Aβ1-40 ratios have been associated with elevated risk of dementia [37]. However, we did not observe significant difference in Aβ1-42/Aβ1-40 ratio between normal control and patients with MCI in this study. One possible explanation is that change in the Aβ1-42/Aβ1-40 ratio is not considerable in the early stages of the disease. Additionally, MCI is a heterogenous clinical syndrome. Some patients may present the early stages of AD, while other may remain stable or even experience symptoms resolution over time [38]. Meanwhile, Aβ1-42 and Aβ1-40 show dynamic changes in the blood [39]. Another study found no significant difference in Aβ1-42/Aβ1-40 ratio among normal control and patients with AD and MCI [40]. Our results also suggests that Aβ1-42 and Aβ1-40 can be employed in combination with other markers to improve the specificity and accuracy of AD.

Non-coding RNA has been implicated in the occurrence and development of AD [41, 42, 43], and serum *miRNA-34a*, *miRNA-34c* and *miRNA-135a* are abnormally expressed in AD. High levels of *miRNA-34a* and *miRNA-34c* and low levels of *miRNA-135a* are risk factors affecting changes associated with the disease [41, 42, 43]. The results of the present study showed that compared with the normal control, the SCD and MCI groups showed significant increases in the serum exosomal *miRNA-34a* and *miRNA-34c* and decreases in *miRNA-135a*expression. These results suggested that exosomal *miRNA-34a*, *miRNA-34c* and *miRNA-135a* influenced the progression of cognitive impairment. *miRNA-34a* and *miRNA-34c* are both involved in the Bcl2 pathway, which regulates cell survival or migration, deacetylase pathway and SIRT1 signaling [44]. Meanwhile, *miRNA-135a* is involved in the biological cascade reaction that can cause neuronal damage, promote neuronal cell apoptosis and disrupt neuroprotective signaling pathways [45]. Overall, changes in the expression levels of these *miRNAs* may promote neuronal injury and cell apoptosis through multiple pathways, leading to varying degrees of cognitive impairment.

The *ApoE* gene is a susceptibility gene to AD and is associated with the occurrence and development of AD. *ApoE* alleles contains ε*2*, ε*3* and ε*4*. *ApoE ε2* exerts a neuroprotective effect, and *ApoE ε2* is a risk factor for AD [46]. *ApoE ε4* carriers have increased risk for AD than *ApoE ε2* carriers [47]. In this study, the *ApoE* genotypes of participants were detected. We found that 10.0%, 50.0% and 75.0% individuals in the normal control, SCD and MCI groups, respectively, are *ApoE ε4* carriers. In the normal control group, ε*3/3* had the highest percentage (62.9%), followed by ε*2/3* (22.9%). Among patients with SCD, the highest percentage was ε*3/3* (31.7%), followed by ε*2/4* and ε*3/4*. Among patients with MCI, the ε*2/4* genotype was the most prevalent (38.3%), followed by ε*3/4* (26.6%). The effects of temperature and climate on *ApoE* genotype have been explored. A study conducted in Kazakhstan showed that the rate of *ApoE ε4* in the northern region (Astana) was 48.3% among patients with AD and was higher than that in the southern region (Almaty, 32.3%) [48]. Eisenberg *et al*. [49] reported that the rate of *ApoE ε4* usually increases with latitude; they hypothesised that metabolic rate may increase in high-latitude cold regions and may require high cholesterol levels. Another hypothesis was that light and vitamin D affect the regional distribution of *ApoE* genotype. High latitudes have short light time and weak UV. Moreover, *ApoE ε4* is associated with elevated levels of serum vitamins. Thus, a high rate of *ApoE ε4* was observed in northern populations that receive less light time [50, 51]. Few studies have analysed the effects of climatic and regional factors on the distribution of *ApoE* genotypes in MCI and SCD. Our study showed that the in cold regions, the distribution of *ApoE* genotypes in MCI and SCD varied. Unfortunately, whether these differences are related to temperature or climate and the underlying mechanisms remain unclear. Subsequent research is needed to explore these gaps.

Our study has some limitations. The collected sample size in the study was small, and thus the conclusions need be further validated using large cohorts. Meanwhile, we did not explore the correlation between changes in the levels of serum markers and brain structure and function. Although SCD may progress to MCI, patients with MCI in this study did not suffer from SCD. Our study failed to identify disease progression based on changes in biomarkers. A longitudinal study could be conducted to analyse the time-varying profiles of biomarkers and brain structure in the progression of SCD to MCI.

## Conclusion

In the early stages of AD, the white matter microstructure was damaged in multiple brain regions, and serum markers including Aβ and exosomal *miRNAs* were altered. In addition, a high proportion of the *ApoE ε4* allele was observed in patients with MCI. These findings may contribute to the early diagnosis and identification of AD patients.

## Availability of Data and Materials

The data used to support the findings of this study are available from the corresponding author upon request.
